# Preparation and Magnetic Properties of Nd/FM (FM=Fe, Co, Ni)/PA66 Three-Layer Coaxial Nanocables

**DOI:** 10.1186/s11671-018-2742-8

**Published:** 2018-10-19

**Authors:** Xiaoru Li, Hongyan Li, Guojun Song, Zhi Peng, Lichun Ma, Chengzhen Meng, Yang Liu, Kaidi Ding

**Affiliations:** Institute of Polymer Materials, School of Materials Science and Engineering, Qingdao University, No. 308 Ningxia Road, Qingdao, 266071 People’s Republic of China

**Keywords:** Nanocable, Magnetic property, Rare earth metal, Electrodeposition

## Abstract

A new preparation method of three-layer coaxial nanocables has been developed in this work. Nd/FM (FM=Fe, Co, Ni)/PA66 three-layer coaxial nanocables were assembled successfully from outer to inner layer by layer. PA66 nanotubes which served as the outer shell were prepared by polymer solution wetting AAO template. Ferromagnetic metals and Nd were deposited into pre-prepared PA66 nanotubes to be served as the middle layer and inner core, respectively. The results show that the structure has effects on the magnetic properties, and the nanocable preparation allows each layer, length, and thickness of the nanocables to be tuned.

## Background

Coaxial nanocable is a special kind of 1D nanostructure in a composite system, which has attracted much interest for its unique structure and properties. Therefore, the nanocables have potential applications in the fields of catalyst, energy storage, photoelectric material, nanobiotechnology, environmental protection, magnetic sensor, and magnetic recording media [1–11]. The development of magnetic recording media is limited by the super-paramagnetic [12] and plane recording limit. Perpendicular recording involves recording data in vertical, three-dimensional columns rather than in two dimensions. To overcome these limitations, either the structure of nanomaterials or the effective anisotropy of the material can be innovated and improved. Ferromagnetic cylinders are suggested as a medium for achieving this objective.

Ferromagnetic cylinders include magnetic multilayer nanowires, nanocables, and ferromagnetic nanotubes and nanowires. Compared with ferromagnetic nanotubes and nanowires, magnetic multilayer nanowires and nanocables have effectively improved magnetic properties [13–16] and extended application fields. Among the various preparation methods, template-based method is one of the most commonly preparation methods. The size, shape, and structural properties of electrodeposited nanocylinders are controlled by the template and electrodeposition parameters. Well known to all, the permanent magnet materials consist of ferromagnetic materials and a rare earth metal. Inspired by these, ferromagnetic nanowires doped with a rare earth element prepared and can change the magnetic properties of composites [17]. To the best of our knowledge, Nd-doped magnetic nanocables have rarely been reported. We have prepared a series of rare earth-doped multilayer nanocable arrays and investigated their magnetic properties [18].

Here, a brief overview of state-of-art nanocable preparation method without using any modifying agent is presented. We employed the anodized aluminum oxide (AAO) template, which has regular channels and a wide range of size and is suitable for nanotube and nanowire and nanocable, to prepare Nd/FM (FM=Fe, Co, Ni)/PA66 triple-layer coaxial nanocables layer by layer. The outermost layer of PA66 nanotubes were fabricated by solution wetting AAO template. The outer shell consisting of a polymer nanotube can keep the inner metal core from being oxidized and eroded and retain the excellent conductivity and magnetism. The middle-layer ferromagnetic nanotubes and internal Nd nanowires were electrodeposited, and in turn, the electrodeposition can control effectively the geometrical structure. The magnetic properties of the coaxial nanocables were studied.

## Methods

### Preparation of PA66 Nanotubes and Working Electrode

Polyamide 66 (PA66) nanotubes can be obtained by wetting the AAO templates (the diameter is about 200 nm, and the thickness is about 60 μm) with 2–6 wt% PA66 formic acid solution. A drop of PA66 solution was placed on a glass slide, and then, a piece of AAO template was covered on the PA66 solution. The PA66 nanotubes were obtained after 40 s. A layer of PA66 film was treated with formic acid to make the PA66 nanotubes open. And then a thin film of Au was sputtered on a side of the PA66/AAO composite membrane to be served as a working electrode.

### Preparation of FM (FM=Fe, Co, Ni)/PA66 Coaxial Nanotubes

Electrolyte solutions were prepared of 0.7 M Ni^2+^, 0.8 M Co^2+^, and 0.8 M Fe^2+^ aqueous solution separately. − 1.0 V/SCE for Ni^2+^, − 1.2 V/SCE for Co^2+^, and − 1.2 V/SCE for Fe^2+^ were employed to prepare Ni, Co, and Fe nanotubes, respectively, in PA66 nanotubes for 15 min to obtain the FM/PA66 double nanotubes.

### Preparation of Nd/FM/PA66 Coaxial Nanocables

1.0 M Nd^3+^ of electrolyte solution was prepared, and then − 2.5 V direct current was input to prepare Nd nanowires into the FM/PA66 coaxial nanotubes for 60 min to form Nd/FM/PA66 coaxial nanocables

**Fig. 1:**
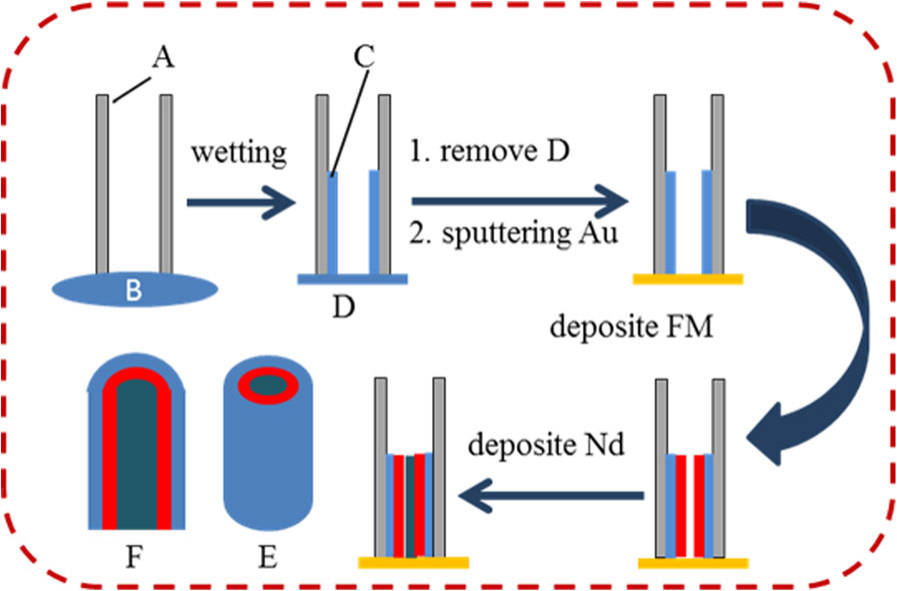
Schematic diagram of preparing tri-layer nanocable: (A) AAO template, (B) polymer solution, (C) polymer nanotube, (D) polymer membrane, (E) and (F) structure and section drawings of nanocable

In the above electrodeposition experiment, a platinum film was used as the counter electrode and an Ag/AgCl electrode in saturated KCl solution as the reference electrode. Figure 1 shows the schematic diagram of preparing three-layer nanocables, as follows:

### Characterization

Scanning electron microscopy (SEM; JEOL JSM-6390LV) and transmission electron microscopy (TEM; CM200-FEG equipped with a GIF) were used to characterize the nanostructures. For TEM measurement, a drop of (5 μL) diluted sample was placed on a copper grid and evaporated prior to observation. The element analysis was identified by X-ray diffraction (XRD; Bruker D8 Advance with a Cu-Kα radiation, *λ* = 1.5418 Å). The magnetization measurements of the FM double nanotubes and Nd/FM/PA66 nanocables were carried out at room temperature on a vibrating sample magnetometer (VSM; Lakeshore 7307).

## Results and Discussion

**Fig. 2:**
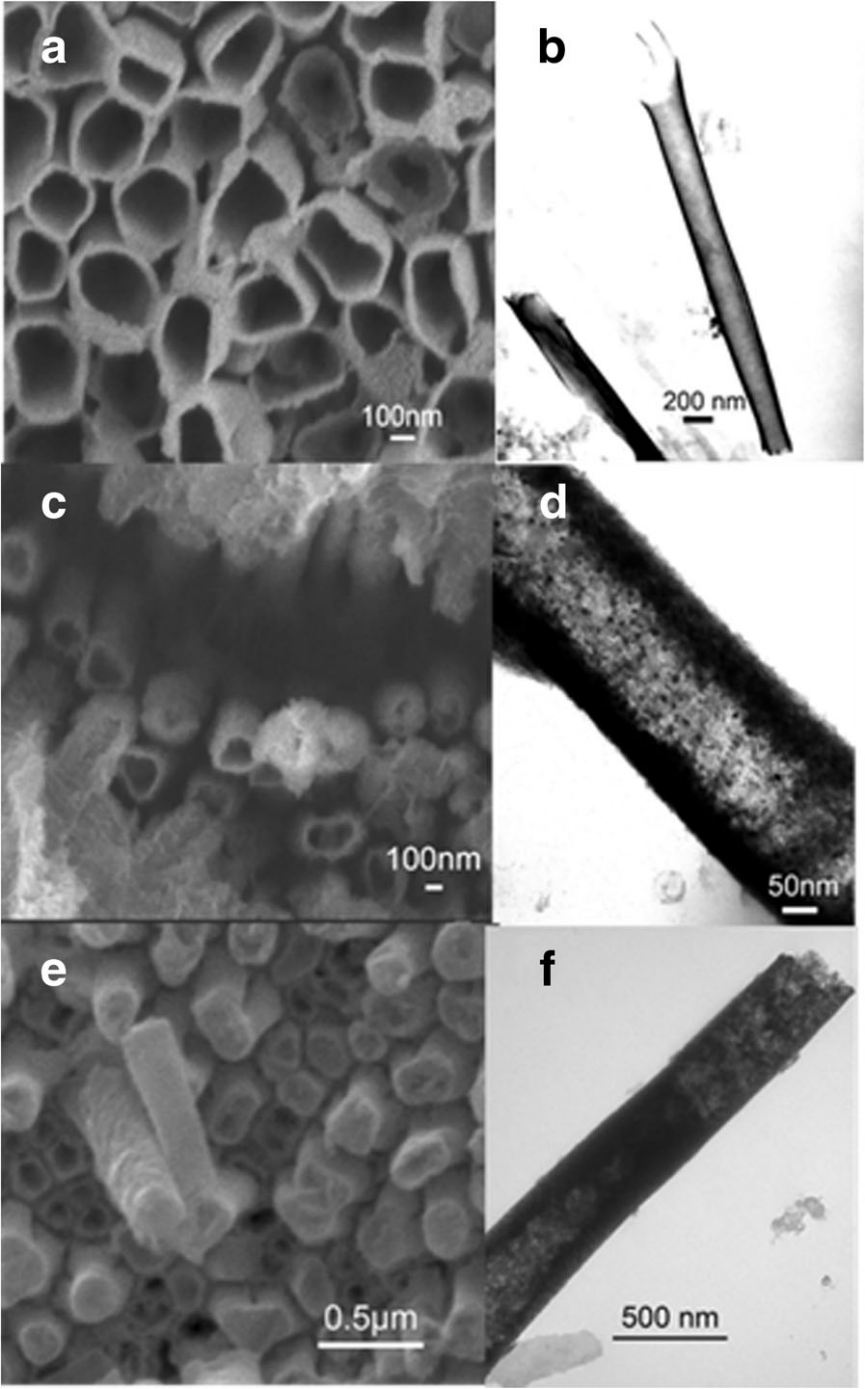
SEM images: **a** Ni/PA66, **c** Co/PA66, **e** Fe/PA66; TEM images: **b** Ni/PA66, **d** Co/PA66, and **f** Fe/PA66

We did a series of condition experiments to ensure better conditions which make PA66 nanotubes and FM nanotubes grow into the same length. The nanostructure of FM/PA66 nanotubes is shown in Fig. 2. As seen from the SEM images shown in Fig. 2a, c, e, FM nanotubes and PA66 nanotubes are nearly of the same length, and the mouths of the nanotubes are almost open. After removing the AAO template, the FM/PA66 nanotubes formed regular arrays. The TEM images further prove the nanostructure of double-layer nanotubes. As seen from Fig. 2b, d, f, the walls of PA66 nanotubes as a sheath are continuous. And the nanoparticles of the FM evenly distributed on the inner wall of PA66 nanotubes. As described in our previous study [19], the diameter of nanoparticles is about 5 nm, and each nanoparticle is regarded as a magnetic domain. A certain amount of FM nanoparticles joined together to form FM nanotubes. Therefore, PA66 nanotubes and FM nanotubes formed double-layer coaxial nanotubes.

**Fig. 3:**
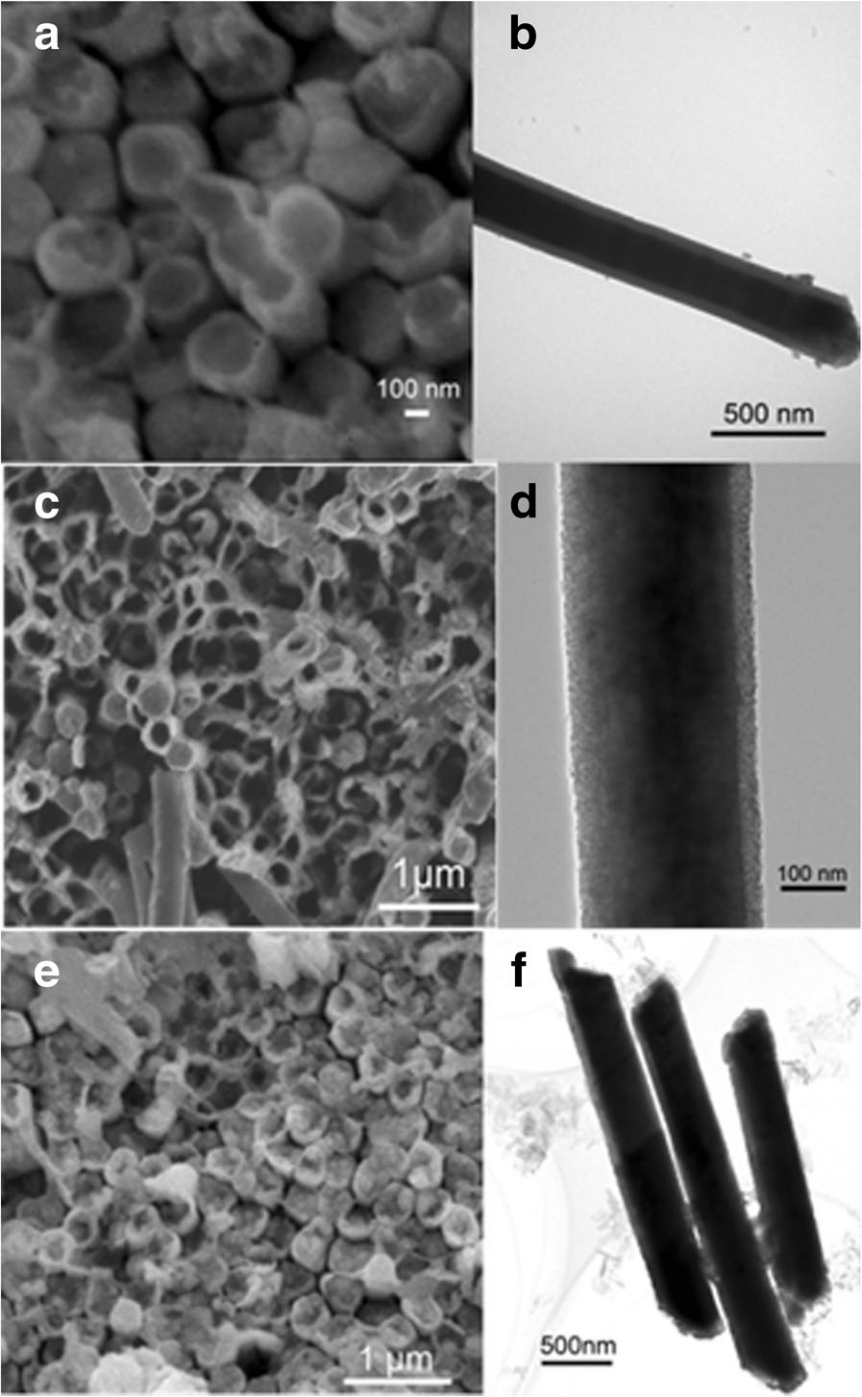
SEM images: **a** Nd/Ni/PA66, **c** Nd/Co/PA66, **e** Nd/Fe/PA66; typical TEM images: **b** Nd/Ni/PA66, **d** Nd/Co/PA66, and **f** Nd/Fe/PA66

Rare earth metals are one of the elements of permanent magnets. Inspired by this, Nd was electrodeposited into the above double-layer nanotubes to make three-layer coaxial nanocables. The morphology of Nd/FM/PA66 nanostructure is shown in Fig. 3. SEM images show that the nanostructures are multilayer and have almost the same length (the size parameters of the nanostructures shown in Table 1). The contrast between the interfaces of polymer and metal is clearly shown in the TEM images. Therefore, the TEM image of the Nd/Ni/PA66 nanocable in Fig. 3b shows that the contrast is clear between the outer layer and the inner layer. The outermost layer is PA66 nanotube with uniform and continuous wall, and the inner layer is composed of Nd and Ni. It is displayed that the inner layer is compact. The contrast cannot be recognized between Nd and Ni because they are all metals. As seen from Fig. 3d, f, it is obvious that the nanostructure is a core/shell structure. Likewise, it is clear for the contrast between the interfaces of PA66 and FM and not clear between the two metals.

**Table 1:** Size parameters, length, and (wall) thickness of nanostructure

Sample	Nd/FM/PA66	PA66	FM	Nd
High	~ 6 μm	~ 6 μm	~ 6 μm	~ 6 μm
Wall thickness	~ 200 nm	~ 50 nm	~ 30 nm	~ 40 nm

**Fig. 4:**
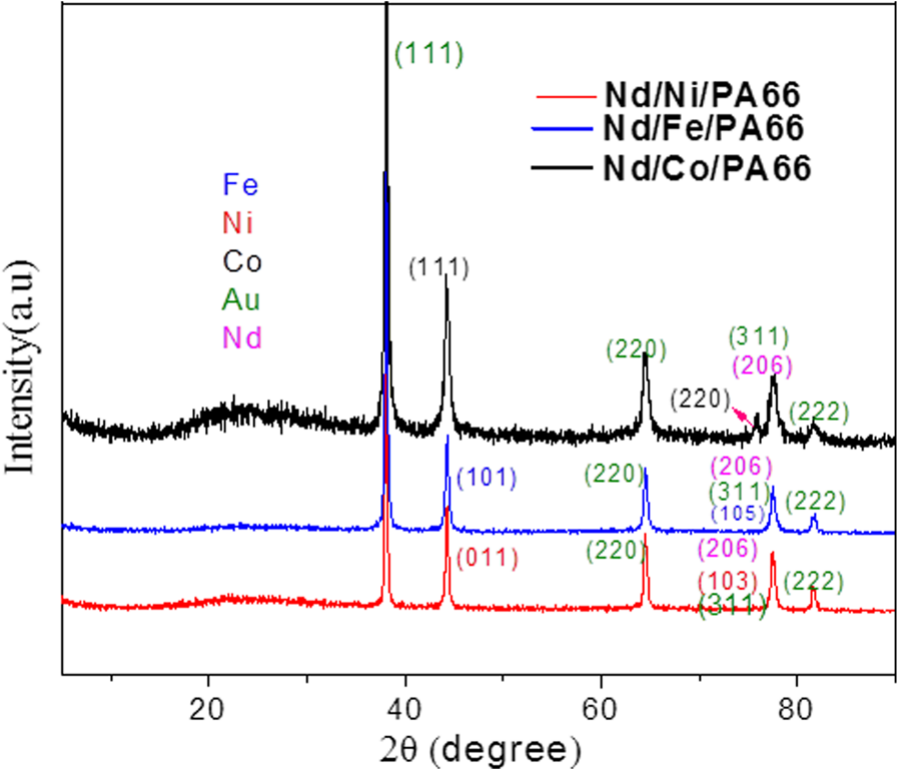
X-ray diffraction patterns of Nd/Ni/PA66, Nd/Co/Pa66, and Nd/Fe/PA66

The X-ray diffraction pattern for the sample is shown in Fig. 4. The distinct diffraction peaks observed at 2*θ* of 44.32° and 75.72° are consistent with the diffraction peak of (111) and (220) crystalline planes of Co, and the diffraction peaks of Fe (101) and Fe (105) correspond with 2*θ* = 44.32° and 77.56°, and the diffraction peaks of Ni (011) and Ni (103) correspond with 2*θ* = 44.32° and 77.56°, respectively. 2*θ* = 77.56° is also a typical diffraction peak of Nd (206). Diffraction peaks (2*θ* of 37.78°, 64.48°, 77.56°, and 81.77°) of Au which were introduced by the sputtered Au film used for electrodeposition are comprehensive, because the value of Au is great, so some of peaks of Au overlap those of Fe and Co and Ni.

**Fig. 5:**
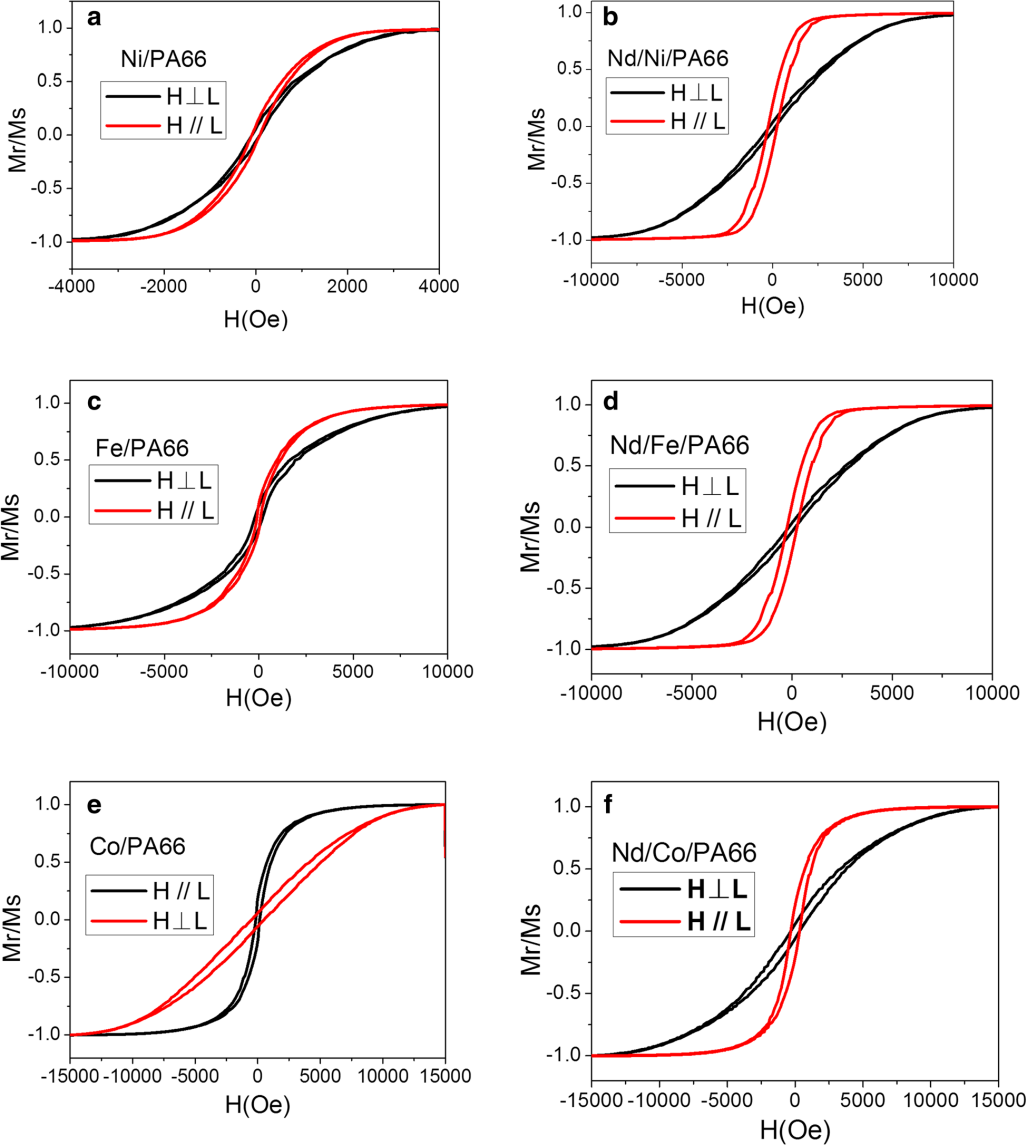
Hysteresis loops: **a** Ni/PA66, **b** Nd/Ni/PA66, **c** Fe/PA66, **d** Nd/Fe/PA66, **e** Co/PA66, and **f** Nd/Co/PA66

The magnetism of all the samples encapsulated in AAO template was measured. AAO templates have a certain amount of antimagnetism and reduced magnetic energy of the samples slightly. Figure 5a–f shows the magnetization hysteresis (M-H) loops of both FM/PA66 nanotubes and Nd/FM/PA66 nanocables. It can be seen that both nanotubes and nanocables have magnetic anisotropy. It is very easy to be understood that the two systems have the same outer diameter, which determines the magnetic anisotropy of the nanotubes and nanocables. The magnetism of nanocables is stronger than that of nanotubes after deposited Nd. This is because Nd, as a typical rare earth metal, possesses a large spin-orbital coupling, when Nd nanoparticles diffused into FM in composite interface and worked together with FM metals, which leads to a synergistic effect and enhances magnetic anisotropy of Nd/FM/PA66 nanocables [20]. According to Fig. 5, the magnetic parameters of the three systems are shown in Table 2. It can be clearly seen that the magnetic parameters of the nanocables such as coercivity and residual magnetization parallel to the long axis are greater than those of vertical direction and nanotubes.

**Table 2:** Magnetic parameters, coercivity (*H*_*c*_), and residual magnetization ratio (*M*_*r*_*/M*_*s*_) of the nanostructures with the field applied parallel (//) and perpendicular (⊥) to the long axis

Sample	*H*_c_^//^	*H*_c_^⊥^	*M*_r_/*M*_s_^//^	*M*_r_/*M*_s_^⊥^
Ni/PA66	94.5	94.5	0.0911	0.0544
Nd/Ni/PA66	246.4	165.3	0.193	0.0365
Fe/PA66	92.39	228.6	0.116	0.0903
Nd/Fe/PA66	235.7	161.2	0.196	0.0394
Co/PA66	185.5	502.8	0.182	0.0565
Nd/Co/PA66	337.8	337.8	0.214	0.0566

## Conclusions

The Nd/FM (FM=Fe, Ni, Co)/PA66 three-layer nanocable arrays have been successfully prepared, respectively. Nd/FM/PA66 nanocables show highly magnetic anisotropy due to the characteristics of rare earth metal and its synergistic effect with FM. The nanocable arrays not only provide a novel magnetic nanostructure but also have potential application in perpendicular magnetic storage and electronic devices.

## Abbreviations

AAO: Anodic aluminum oxide
FM: Fe, Co, Ni
M-H: Magnetization hysteresis loops
PA66: Polyamide 66
SEM: Scanning electron microscope
TEM: Transmission electron microscope
XRD: X-ray diffraction

## References

[1] Tang YL, Tian JL, Malkoske T, Le WJ, Chen BD (2017). Facile ultrasonic synthesis of novel zinc sulfide/carbon nanotube coaxial nanocables for enhanced photodegradation of methyl orange. J Mater Sci.

[2] Zhang Y, Tang YK, Gao SS, Jia DZ, Ma JH, Liu L (2017). Sandwich-like CNT@Fe_3_O_4_@C coaxial nanocables with enhanced lithium-storage capability. ACS Appl Mater Interfaces.

[3] Wang Y, Qu QT, Li GC, Gao T, Qian F, Shao J, Liu WJ, Shi Q, Zheng HH (2016). 3D interconnected and multiwalled carbon@MoS2@Carbon hollow nanocables as outstanding anodes for Na-Ion batteries. Small.

[4] Lu Y, Wang K, Li JW, Li YH, Zhang W, Sui ML (2016). Oxidative corrosion mechanism for Ag@C coaxial nanocables in radiolytic water. J Phys Chem C.

[5] Liu WN, Chen JH, Yang T, Chou KC, Hou XM (2016). Enhancing photoluminescence properties of SiC/SiO2 coaxial nanocables by making oxygen vacancies. Dalton T.

[6] Ullah MH, Ha CS (2016). In situ prepared polypyrrole-Ag nanocomposites: optical properties and morphology. J Mater Sci.

[7] Zhang M, Wang YT, Zhang YW, Ding L, Zheng J, Xu JL (2016). Preparation of magnetic carbon nanotubes with hierarchical copper silicate nanostructure for efficient adsorption and removal of hemoglobin. Appl Surf Sci.

[8] Liu QF, Gao CX, Xiao JJ, Xue DS (2003). Size effects on magnetic properties in Fe_0.68_Ni_0.32_ alloy nanowire arrays. J Magn Magn Mater.

[9] Yanagishita T, Nishio K, Masuda H (2008). Antireflection polymer hole array structures by imprinting using metal molds from anodic porous alumina. Appl Phys Express.

[10] Cernea H, Vasile BS, Surdu VA, Trusca R, Craciun F, Falassi C (2018). Synthesis and characterization of CoFe2O4/BNT-BT0.08 core–shell nanotubes by a template based sol-gel method. Ceram Int.

[11] Kalidasan V, Liu XL, Herng TS, Yang Y, Ding J (2016). Bovine serum albumin-conjugated ferrimagnetic iron oxide nanoparticles to enhance the biocompatibility and magnetic hyperthermia performance. Nano-Micro Lett.

[12] Han XF, Shamaila S, Sharif R, Chen JY, Liu HR, Liu DP (2009). Structural and magnetic properties of various ferromagnetic nanotubes. Adv Mater.

[13] Shakya P, Cox B, Davis D (2012). Giant magnetoresistance and coercivity of electrodeposited multilayered FeCoNi/Cu and CrFeCoNi/Cu. J Magn Magn Mater.

[14] Béron F, Carignan LP, Ménard D, Yelon A (2008). Magnetic behavior of Ni/Cu multilayer nanowire arrays studied by first-order reversal curve diagrams. IEEE Trans Magn.

[15] Li XR, Wang YQ, Song GJ, Peng Z, Yu YM, She XL, Sun J, Li JJ, Li PD, Wang ZF, Duan XF (2010). Fabrication and magnetic properties of Ni/Cu Shell/Core nanocable arrays. J Phys Chem C.

[16] Li XR, Yang C, Han P, Zhao QP, Song GJ (2016). Facile synthesis and magnetic study of Ni@polyamide 66 coaxial nanotube arrays. J Magn Magn Mater.

[17] Wang Q, Yang XW, Li SM, Yan C, Zhang H, Yang H (2008). Hydrothermal-induced oriented growth of Fe–Co alloy and Sm^3+^-substituted magnetite nanowire composites. J Magn Magn Mater.

[18] Li XR, Wang XX, Ma LC, Peng Z, Yang C, Han P, Li HY, Miao YC, Long YZ, Song GJ (2018). Effect of each layer on anisotropic magnetic properties of Nd/Fe/polyamide 66 three-layer coaxial nanocables. ACS Omega.

[19] Li XR, Wang YQ, Song GJ, Peng Z, Yu YM, She XL, Li JJ (2009). Synthesis and growth mechanism of Ni nanotubes and nanowires. Nanoscale Res Lett.

[20] Sepehri-Amin H, Ohkubo T, Hono K (2013). The mechanism of coercivity enhancement by the grain boundary diffusion process of Nd-Fe-B sintered magnets. Acta Mater.

